# Effects of intrinsic and extrinsic factors on ruminating, grazing, and bedding time in bighorn sheep (*Ovis canadensis*)

**DOI:** 10.1371/journal.pone.0206664

**Published:** 2018-10-29

**Authors:** Muyang Wang, Joana Alves, Meghan Tucker, Weikang Yang, Kathreen E. Ruckstuhl

**Affiliations:** 1 CAS Key Laboratory of Biogeography and Bioresources in Arid Land, Xinjiang Institute of Ecology and Geography, Urumqi, China; 2 Centre for Functional Ecology (CFE), Department of Life Sciences, University of Coimbra, Coimbra, Portugal; 3 Department of Biological Sciences, University of Calgary, Calgary, Canada; The University of Sydney, AUSTRALIA

## Abstract

Rumination is the repeated process of regurgitation of a food bolus, followed by chewing, swallowing, and regurgitation, which enhance nutrient assimilation. Time spent in lateral recumbency (i.e., bedded, lying) has often been used as a proxy for time spent ruminating due to difficulties of observing detailed rumination behavior in the field. The actual proportion of time spent ruminating, or other activities, will in turn be affected by the age and sex of an individual but also with changes in food quality. We studied the effects of intrinsic and extrinsic factors on time spent ruminating, bedding, proportion of bedding time spent ruminating, and grazing of individually marked bighorn sheep (*Ovis canadensis*). Our results show that bighorn sheep spent more time ruminating and less time grazing in summer and autumn. Overall, females spent less time ruminating, and more time grazing than males. Bighorn sheep decreased their time spent ruminating with increasing acid detergent fiber (ADF) content in the forage. Age influenced the time spent grazing, bedded and proportion of bedded time spent ruminating. Older sheep not only increased their bedding time but also their time spent bedded without ruminating compared to younger individuals. The proportion of time spent grazing was also affected by age, with a decrease in the proportion of time spent grazing with increasing age. Our results suggest that these four behaviors are plastic and variable. We thus conclude that bedding time does not reflect time spent ruminating but that the latter is affected by both intrinsic and extrinsic factors.

## Introduction

Rumination is the mechanical breakdown of ingested food through bouts of repeated regurgitation of boluses that bring food back into the animal’s mouth to then be re-chewed and swallowed. This process allows the degradation of forage into small particles, and thus can enhance nutrient assimilation[1–3].

Rumination is considered a plastic behavior, as both intrinsic and extrinsic factors may influence chewing parameters[2,4]. Among all intrinsic factors, sex and age, which are related to body size and thus size of the rumen, are the most important factors [2,5] affecting nutrient absorption of different quality forage[6]. In size-dimorphic animals, the smaller females select higher quality forage due to their higher metabolic energy requirements per unit of body mass, their smaller gut capacity and lower digestive efficiency, while the bigger males need more food overall[7,8]. Mastication efficiency is crucial to digestion processes in ruminants. The degree of tooth wear increases with age[2]. Therefore, the teeth’s ability to chew forage should increase with age until they are fully developed, and are then expected to be at their maximum efficiency until gradually decreasing with age. We thus expect older individuals to alter their time spent foraging and ruminating to compensate for a decrease in mastication efficiency.

Animals exhibit different ruminating patterns depending on the quality of forage in their habitat[9]. Poor-quality forage can be defined as containing a large proportion of cellulose and lignin, which means ruminants will need more time to process it. High-quality forage, however, is much easier to chew and digest, and thus requires a shorter time to process[10]. For instance, bighorn sheep spent less time processing high-quality forage by increasing their chewing rate (number of chews/second) per bolus of regurgitated food[6]. Foraging and ruminating patterns of ruminants are thus thought to be affected by the seasonal variation of quality of the consumed forage[11].

Bighorn sheep are typically sexually segregated outside the rut but it has been argued that, at low population sizes, the anti-predator benefits from forming larger, albeit mixed-sex, groups can outweigh the cost of synchronizing activities with the opposite sex [12]. Ruckstuhl reported that individuals in mixed-age groups or in mixed-sex (henceforth called mixed) groups synchronized their behavior to that of the majority and, therefore, departed from their optimal activity budgets[13]. These potential synchrony costs could limit the opportunities for individuals to compensate for time lost from grazing or ruminating when in mixed groups. While animals cannot compensate for time lost foraging or ruminating in mixed groups, we argue that they can compensate for these potential “costs” by changing bite rates, chewing rates, and the proportion of time spent ruminating during a bedding bout. Therefore, bighorn sheep are expected to change their activity budgets depending on the group type they are in[14].

Studies on “group size” effects usually focus on the trade-offs between time spent feeding and being vigilant. Animals in larger groups should be feeding longer [15,16], and thus have more forage to process in their rumen. Penning et al. empirically tested whether group size affected grazing time in penned domestic sheep (*Ovis aries*) by experimentally increasing group size[17]. Individuals in small groups (1–3 individuals) spent less time foraging than when in larger groups (> 4 individuals). However, a further increase in group size from 4 to 5, 6, and up to 10 did not lead to a further increase in grazing time. Group size thus needs to be taken into consideration when investigating activity budgets.

In this paper, we investigated the proportion of time bighorn sheep spent ruminating, grazing, bedded, and the proportion of bedding time spent ruminating, to explore potential links between intrinsic and extrinsic factors and these four behaviors. According to the theories and hypotheses outlined above, we make the following predictions: (1) Bighorn sheep decrease their proportion of time spent ruminating in summer, when the quality of forage is better (lower ADF levels), and increase ruminating time in winter, when the quality of forage is poorer (higher ADF levels). (2) Rumination time will be affected by group type, with mixed groups presenting intermediate behaviors. (3) Group size positively affects foraging and ruminating time. (4) Older individuals increase their time spent ruminating to compensate for a decrease in mastication efficiency. (5) Sex affects activity budgets, with males spending more time bedded than grazing, while females spend more time grazing than bedded.

## Materials and methods

### Study area and animals

Sheep River Provincial Park (SRPP) is located in the foothills of the Rocky Mountains in southwestern Alberta (50°N, 114°W; between 1,420–1,740 m of elevation). The area is composed of large hills with south-facing meadows which are interspersed with aspen stands (*Populus tremuloides*) and coniferous forests (mostly white spruce (*Picea glauca*) and lodgepole pine (*Pinus contorta*)[18]. Bighorn sheep have been ear-tagged since 1981, and are individually identifiable[19]. We know the exact age of all males or count horn annuli on unmarked individuals[20]. The age of most (99%) females is known, because they were caught as lambs or before the age of 4, when they lose their last 2 milk teeth (lower lateral incisors) and complete their adult dentition[21]. Males were considered adults at 4 years of age and ewes at 3 years of age[18]. Bighorn sheep at SRPP are sexually segregated outside the breeding season[18]. Female or nursery groups consist of ewes, lambs and subadults (< 4 years old) of both sexes. Adult males form male or bachelor groups, that typically consist of rams, 4 years and older. Mixed groups include at least one adult male and female. Bighorn sheep females migrate to the mountains to give birth to their lambs at the end of May and the first 2 weeks of June, and only sporadically visit SRPP with their lambs, until they all migrate back in August/early September[18]. Because of this migration, we could not collect data on female groups in July.

### Behavioral observations

To avoid observer bias, all data was collected by only one observer, Ben Curry, who was trained and hired by K. E. Ruckstuhl. The sheep are habituated to humans and can be easily observed at a short distance. Observations of rumination behavior are therefore possible using spotting scopes (Bushnell, 25–60×zoom) or binoculars (Leica, 10 × 42), while keeping an adequate distance (~50 meters). Bedding was defined as periods when an individual lay down in sternal recumbency and with their head up or with head resting on the ground. Rumination is defined as the process of passing previously ingested food (AKA a bolus) up the esophagus into the mouth, chewing and insalivating the bolus repeatedly, and finally swallowing it. This process is visible to the naked eye in our study population.

The behaviors of bighorn sheep were recorded using the focal animal sampling method [22], from May 2005 to April 2006. We did not collect data during the rut (late November—late December), because male bighorn sheep activity budgets are significantly altered at that time of year: males go through hypophagia, and invest most of their time in rutting activities[23]. All groups, and individuals therein, were selected randomly and observed continuously between a minimum of 5.5 hours, and a maximum of 12.5 hours with a mean of 8 hours per day, during which the exact start and stop time of rumination, grazing and bedding time was recorded to the nearest minute using a stop watch. More than 30 individual animals were observed, and all their behaviors recorded, each month, except during the rut.

For each focal individual (experimental unit), we recorded their ID, sex and age, and environment (group type, group size, season, and ADF content of forage). Bighorn sheep at SRPP bed down to ruminate, and we did not observe any animals ruminating while standing for more than a minute. The total proportion of bedded time spent ruminating was therefore calculated by dividing time (in minutes) spent ruminating by time spent bedded for each focal individual.

A group was defined as animals that were in close proximity to each other, and the distance to the nearest neighbor did not exceed 50 meters. A much more recent study by McDougall and Ruckstuhl (2018), actually showed that mimicry of vigilance behaviour between nearest neighbours, for example, dropped significantly when interindividual distances were ≥ 10 meters [24]. Nevertheless, coordinated movement might still be possible at larger distances, up to 50 meters. The group composition and size did not change within a given observation day. However, bighorn sheep live in a fission-fusion society and the composition and size of groups changes with time[13,18]. Overall, we conducted a total of 302 observations of > 5-hour periods on 65 individuals, representing the sexes and seasons fairly equally.

### Environmental variables

Since preliminary results, testing the effect of month or season on bighorn activity budgets, were the same, we grouped the data into four seasons: spring (April–May), summer (June–August), autumn (September–November) and winter (January–March). Forage quality in our study area is best in summer, and poorest in winter, which was measured via the acid detergent fiber (ADF) concentration in the forage; an increase in ADF is negatively associated with forage quality[6,18,25]. The data used in the present study were collected from the same individuals, locations, and time periods as published in Moquin et al.[6].

### Data analysis

The statistical analyses were performed using IBM SPSS Statistics 23 (IBM Corporation, New York, USA). To test our predictions, linear mixed models (LMM) were used to analyze the effects of potential intrinsic (sex and age; fixed factors) and extrinsic factors (group type, group size, season and forage quality; fixed factors) on the proportion of time spent ruminating, bedded, proportion of bedded time spent ruminating, and grazing (response variables) (Model 1). We tested the effect of forage quality (ADF) (an increase in ADF is negatively associated with forage quality) (Model 2) on the proportion of time spent in each behavior separately, due to its collinearity with season. Animal ID and date of observation were both included as random factors in all the analyses, to account for any potential pseudo-replication resulting from multiple observations of the same individuals over time. Date was added in lieu of group ID, since group composition changed over the course of several days. The independence of predictor variables was confirmed through correlation analyses and variance inflation factors[26]. We checked for any potential interactions between our independent variables. Only the interaction between season and sex was significant when analyzing the proportion of time spent ruminating while bedded, and was thus retained in the models. Since response variables are proportions of time spent performing each behavior (values restricted between 0 and 1) a logit transformation was done to achieve a normal distribution and to reduce heterogeneity[27]. To increase robustness of the estimates and deal with any potential constrains in terms of sample size and unbalanced data, a Satterthwaite approximation to the degrees of freedom was used. After fitting the LMMs, model validation was performed on the residuals by checking heteroscedasticity and normality[28]. The results are expressed as estimated means from the LMM ± standard error (SE) and 95% confidence intervals (CI), unless otherwise stated. All statistical tests were considered significant when P < 0.05.

### Ethics statement

University of Calgary’s Animal Care Committee approved the research. No further approval by an Ethics Committee was required, as behavioral observations at a distance were non-invasive.

## Results

### Rumination

The proportion of time spent ruminating differed between seasons (F_3,31_ = 4.440; P = 0.01) (Table 1), with higher rumination time in autumn (β = 0.48±0.135; P < 0.01) and summer (β = 0.35±0.148; P = 0.03), compared to winter. Regarding intrinsic factors, sex was found to significantly affect rumination time (F_1,143_ = 5.536; P = 0.02), with females spending less time ruminating (β = -0.20±0.083; P = 0.02) than males (S1 Fig). Rumination was not affected by age (F_1,100_ = 2.276; P = 0.14) or group type (F_2,42_ = 2.116; P = 0.13), but was negatively affected by group size (F_1,80_ = 16.916; P < 0.01; β = -0.02±0.004) (Table 2).

**Table 1:** Proportion of time bighorn sheep spent in different activities by each of the seasons and each sex; estimated using linear mixed models.

	Season									Sex				
	Spring		Summer		Autumn		Winter		P	Male		Female		P
	(N = 52)		(N = 65)		(N = 76)		(N = 109)			(N = 222)		(N = 80)		
	LSMean	SEM	LSMean	SEM	LSMean	SEM	LSMean	SEM		LSMean	SEM	LSMean	SEM	
Rumination	0.24	0.008	0.27	0.006	0.32	0.005	0.17	0.004	0.01	0.26	0.005	0.19	0.007	0.02
Bedding	0.16	0.015	0.30	0.010	0.19	0.009	0.09	0.008	<0.01	0.20	0.007	0.10	0.011	0.15
Bedded time spent ruminating	0.63	0.020	0.47	0.012	0.63	0.010	0.69	0.014	0.05	0.59	0.009	0.70	0.017	0.57
Grazing	0.52	0.021	0.33	0.013	0.38	0.010	0.66	0.012	<0.01	0.44	0.011	0.65	0.017	<0.01

LSMean is the mean estimated from each linear mixed model, and SEM is the estimated standard error of the mean. P is the probability value.

**Table 2:** Two models on the potential association of intrinsic and extrinsic factors with the proportion of time bighorn sheep spent ruminating.

Term	Coefficient (β)	SE	t	P
*Model 1*				
Intercept	-1.03	0.150	-6.837	<0.01
Season				
Spring	0.23	0.147	1.579	0.13
Summer	0.35	0.148	2.367	0.03
Autumn	0.48	0.135	3.589	<0.01
Winter	0*			
Sex				
Female	-0.20	0.083	-2.353	0.02
Male	0*			
Age	0.01	0.009	1.509	0.14
Group size	-0.02	0.004	-4.113	<0.01
Group type				
Nursery	0.31	0.167	1.873	0.07
Male group	-0.02	0.126	-0.182	0.86
Mixed group	0*			
*Model 2*				
Intercept	0.55	0.640	0.855	0.40
Sex				
Female	-0.23	0.083	-2.742	0.01
Male	0*			
Age	0.01	0.009	1.573	0.12
Group size	-0.02	0.004	-5.311	<0.01
Group type				
Nursery	0.36	0.183	1.948	0.06
Male group	-0.08	0.144	-0.529	0.60
Mixed group	0*			
Forage quality (ADF)	-0.03	0.014	-2.039	0.05

*Reference category. SE is the standard error of the model coefficient.

Acid detergent fibre marginally influenced the proportion of time spent ruminating (F_3,37_ = 4.158; P = 0.05). Higher acid detergent fiber values were associated with a decrease in the proportion of time spent ruminating (β = -0.03±0.014) (Table 2).

### Bedding

The proportion of time spent bedded differed across seasons (F_3,43_ = 5.215; P < 0.01), and was affected by group type (F_2,60_ = 3.330; P = 0.04) and age (F_1,80_ = 5.115; P = 0.03) (Table 3). Male groups spent more time bedded than nursery and mixed groups in all seasons. In mixed groups, males and females did not differ in the proportion of time spent bedded (F_1,132_ = 2.119; P = 0.15) (Table 1). Group size did not affect the time spent bedded (F_1,113_ = 0.027; P = 0.87). Bedding time of bighorn sheep increased with age (Table 3 and S2 Fig).

**Table 3:** Two models on the potential association of intrinsic and extrinsic factors with the proportion of time bighorn sheep spent bedded.

Term	Coefficient (β)	SE	t	P
*Model 1*				
Intercept	-3.10	0.368	-8.439	<0.01
Season				
Spring	0.93	0.338	2.759	0.01
Summer	1.31	0.340	3.864	<0.01
Autumn	0.79	0.309	2.568	0.01
Winter	0*			
Sex				
Female	-0.34	0.231	-1.456	0.15
Male	0*			
Age	0.06	0.027	2.262	0.03
Group size	0.00	0.009	-0.165	0.87
Group type				
Nursery	-0.20	0.384	-0.527	0.60
Male group	0.65	0.290	2.239	0.03
Mixed group	0*			
*Model 2*				
Intercept	1.85	1.319	1.403	0.17
Sex				
Female	-0.34	0.229	-1.506	0.14
Male	0*			
Age	0.06	0.027	2.216	0.03
Group size	-0.01	0.009	-0.759	0.45
Group type				
Nursery	-0.14	0.376	-0.374	0.71
Male group	0.60	0.305	1.970	0.05
Mixed group	0*			
Forage quality (ADF)	-0.10	0.028	-3.504	<0.01

*Reference category. SE is the standard error of the model coefficient.

Forage quality also influenced the proportion of time spent bedded (F_1,45_ = 12.281; P < 0.01; β = -0.10±0.028), with higher ADF values leading to a lower proportion of time spent bedded (Table 3 and S2 Fig).

### Proportion of bedded time spent ruminating

The proportion of bedded time spent ruminating showed a significant interaction between season and sex (F_3,226_ = 3.405; P = 0.02), with females spending a higher proportion of bedded time ruminating, in spring and summer (Table 4 and S3 Fig).

**Table 4:** Two models on the potential association of intrinsic and extrinsic factors with the proportion of bedded time bighorn sheep spent ruminating.

Term	Coefficient (β)	SE	t	P
*Model 1*				
Intercept	0.96	0.160	5.960	<0.01
Season				
Spring	-0.69	0.221	-3.124	<0.01
Summer	-0.84	0.199	-4.196	<0.01
Autumn	-0.28	0.188	-1.460	0.15
Winter	0*			
Sex				
Female	0.02	0.166	0.096	0.92
Male	0*			
Age	-0.04	0.016	-2.257	0.03
Season×Sex				
Spring×Female	0.87	0.282	3.086	<0.01
Spring×Male	0*			
Summer×Female	0.43	0.433	0.985	0.33
Summer×Male	0*			
Autumn×Female	0.03	0.287	0.096	0.92
Autumn×Male	0*			
Winter×Female	0*			
Winter×Male	0*			
*Model 2*				
Intercept	-1.17	0.817	-1.427	0.16
Sex				
Female	0.08	0.150	0.522	0.60
Male	0*			
Age	-0.03	0.017	-2.025	0.05
Group size	-0.01	0.006	-0.757	0.45
Group type				
Nursery	0.25	0.233	1.066	0.29
Male group	-0.31	0.197	-1.587	0.12
Mixed group	0*			
Forage quality (ADF)	0.05	0.017	2.732	0.01

*Reference category. SE is the standard error of the model coefficient.

With increasing age, sheep decreased their proportion of bedded time spent ruminating (F_1,89_ = 5.096; P = 0.03; β = -0.04±0.016) (Table 4 and S3 Fig). The proportion of bedded time spent ruminating also significantly changed with ADF content (F_1,46_ = 7.464; P = 0.01), increasing as ADF increased (β = 0.05±0.017), particularly for males (Table 4 and S3 Fig).

### Grazing

Bighorn sheep females and males showed marked differences in the proportion of time spent grazing (F_1,140_ = 39.386; P < 0.01; Table 1), with females spending more time grazing than males (β = 0.54±0.086) across seasons, except for autumn (S4 Fig). Age was a significant intrinsic factor (F_1,93_ = 25.005; P < 0.01), with a decrease in time spent grazing with increasing age (β = -0.05±0.009; S4 Fig). The proportion of time sheep spent grazing was significantly different across seasons (F_3,28_ = 5.330; P < 0.01), with grazing proportions in summer (β = -0.98±0.248) and autumn (β = -0.60±0.225) being lower compared to winter (Table 1). Grazing proportion was also significantly affected by group size (F_1,209_ = 49.583; P < 0.01), with grazing increasing with increasing group size (β = 0.036±0.005) (Table 5).

**Table 5:** Two models on the potential association of intrinsic and extrinsic factors with the proportion of time bighorn sheep spent grazing.

Term	Coefficient (β)	SE	t	P
*Model 1*				
Intercept	-0.14	0.214	-0.634	0.53
Season				
Spring	-0.43	0.248	-1.752	0.09
Summer	-0.98	0.248	-3.938	<0.01
Autumn	-0.60	0.225	-2.682	0.01
Winter	0*			
Sex				
Female	0.54	0.086	6.276	<0.01
Male	0*			
Age	-0.05	0.009	-5.000	<0.01
Group size	0.036	0.005	7.042	<0.01
Group type				
Nursery	-0.18	0.275	-0.670	0.51
Male group	0.13	0.180	0.729	0.47
Mixed group	0*			
*Model 2*				
Intercept	-4.28	0.885	-4.835	<0.01
Sex				
Female	0.55	0.086	6.433	<0.01
Male	0*			
Age	-0.05	0.009	-5.021	<0.01
Group size	0.04	0.005	7.514	<0.01
Group type				
Nursery	-0.16	0.259	-0.611	0.55
Male group	0.21	0.182	1.130	0.26
Mixed group	0*			
Forage quality (ADF)	0.08	0.019	4.409	<0.01

*Reference category. SE is the standard error of the model coefficient.

As mentioned earlier, when acid detergent fiber values increase forage quality decreases. Our results showed that forage quality also had a significant effect on the time spent grazing in both sexes (F_1,140_ = 19.435; P < 0.01), with an increase in time spent grazing as acid detergent fiber (ADF) content increased (poor-quality forage) (β = 0.08±0.019) (Table 5 and S4 Fig).

## Discussion

Our results clearly indicate that the time spent grazing, ruminating, bedding and proportion of bedded time spent ruminating are plastic behaviors that are affected by intrinsic and extrinsic factors. Forage quality (ADF) and season influenced all four behaviors. Ruminating and grazing time differed between the sexes and were affected by group size. Age affected the proportion of time spent grazing, bedded, the proportion of bedded time spent ruminating, and group type only had an effect on the proportion of time spent bedded.

High-quality forage (or low ADF content) is much easier to chew and digest, and thus can be processed more rapidly than low-quality vegetation (high ADF content). Accordingly, ruminants should spend less time ruminating with increasing forage quality (low ADF)[9, 10]. Contrary to this prediction, we found that bighorn sheep decreased their proportion of time spent ruminating with increasing ADF content. Moquin et al. reported that the same bighorn sheep population increased their chewing rates and decreased their bolus processing time with an increase in ADF content[6]. Considering these results, it seems reasonable to propose that bighorn sheep, and likely other size-dimorphic ruminants use several different behaviors to optimize nutrient extraction: as acid detergent fiber content increases they can either increase the number of chews per bolus, increase foraging time, or process more boluses per bedding bout. Our study confirmed that changes in forage quality affected bighorn sheep behavior, as reported by Moquin et al.[6], but instead of increasing rumination time (as we predicted), they increased their grazing time.

Forage quality also plays an important role when considering how sex and body size affect the proportion of time spent in different behaviors, particularly in species with a large dimorphism in body mass[29,30]. Sexual differences in activity budgets was proposed as one of the proximate factors responsible for sexual segregation[31]. When food quality is lower, larger individuals (usually males) have longer retention times, which allows extracting more nutrients from the forage[32,33,34], and requiring longer rumination times compared to females[35,36]. In agreement with these ideas, our study found that bighorn sheep males spent more time ruminating than females for most of the year, except in summer, when forage quality was high[6]. Males and females did not differ in the quality of forage they consumed[18,37]. However, males only moved one fourth of the distance females travelled per day[18], and spent more time bedded and less time forging than females, when food quality was higher.

Regarding season, as expected all behaviors were influenced by this extrinsic factor, which may be explained by changes in temperature, forage quality, daylight duration, or even the bighorn sheep life stages that occur in each of the studied seasons. However, since the results obtained regarding season followed the same trend of the ones obtained for forage quality (ADF), seems that the quality of the forage plays a crucial role in explaining the proportion of time spent ruminating, bedded and grazing by bighorn sheep.

Bighorn sheep are alternating grazing and bedding throughout the day and only bed down for the night at dusk in SRPP[18]. They are known to be diurnal feeders[38] and are thus not expected to graze at night. To corroborate this idea, Ruckstuhl (unpublished) conducted random spotlight searches at night throughout the year to see whether any sheep were grazing or moving. The only time of year when animals were observed being active at night was during the rut, when many males but also some females were on their way towards or from the mountains. Another indication that sheep were not foraging or moving during the night, was evident when looking at an individual’s dung pile that had accumulated over night. They were much larger than dung pile found at daytime bedding sites. However, we could not conduct focal observations at night and we could therefore not assess or control for any time spent ruminating at night. We hence cannot exclude the possibility that some individuals compensated for lost rumination time during the day, by allocating more time to ruminating at night. However, if they are not active at night and thus do not forage, then rumination time will still be limited by rumen size, digestive efficiency and gut capacity, which in turn is determined by intrinsic and extrinsic factors[9].

There are several advantages to living in groups for ungulates, such as decreased vigilance and increased foraging time, with increasing group size[12,18]. Individuals in larger groups were expected to increase their ruminating time due to a decrease in vigilance and an increase in foraging time, which in turn affects the amount of forage in the rumen needing to be processed[14]. Individuals in larger groups increased their bolus processing time, but an opposite trend was found for chewing rate, which can be explained by the expected decrease in vigilance in larger groups[6]. According to our findings, grazing and rumination were affected by group size, with animals spending more time grazing in larger groups, and less time ruminating. Bighorn sheep could process forage while maintaining the collective vigilance levels and group cohesion by increasing chewing effort, instead of increasing time spent ruminating. Ruckstuhl previously showed an effect of group type on activity budgets, where individuals in nursery groups had significantly different activity budgets compared to individuals in bachelor groups, whereas mixed groups showed intermediate activity budgets[13]. To maintain group cohesion, individuals may synchronize activities at the expense of foraging or bedding time, which seems to be happening in our population of bighorn sheep[12,13]. When in mixed groups, males and females spent a similar proportion of time bedded, but their activity budgets differed significantly when in unisex groups. However, and contrary to what we expected, group type only affected the proportion of time individuals spent bedded but had no effect on time spent ruminating or grazing.

Bighorn sheep modified their time spent bedded, proportion of bedded time spent ruminating, and grazing according to their age (which is correlated with body size or mass). Older individuals increased bedding time, and decreased the proportion of grazing-, and bedded-time spent ruminating, compared to younger ones. The decrease in time spent foraging and the corresponding increase in time spent bedded with age may be due to a lower relative metabolic rate of older individuals[4]. Older individuals therefore may decrease rumination due to a decrease in the amount of forage in the rumen needing to be processed[14]. This means that older animals will need the same proportion of time to ruminate a smaller amount of food, if teeth get worn down with age, as we predicted.

Time spent bedded is frequently treated as a proxy for time spent ruminating, mostly due to the difficulties of observing rumination behavior in the field [2,4,39,40]. In our study on individually marked bighorns, 67.70% (a minimum of 0% and a maximum of 100%) of a female’s time spent bedded was spent ruminating, whereas only 56.58% (a minimum of 15.79% and a maximum of 100%) of a male’s time was spent ruminating. This variance in the proportion of bedded time spent ruminating reflects the heterogeneity of ruminating behavior in bighorn sheep and possibly other ruminants. We thus suggest that bedding time alone should not be used as a proxy for rumination time, because this may lead to incorrect conclusions about the real amount of time spent ruminating, which in turn can mask sexual differences in rumination versus bedding time.

## Conclusions

Overall, our results show that ruminating, bedding, the proportion of bedded time spent ruminating, and grazing times are plastic behaviors constrained by both intrinsic and extrinsic factors, such as season, sex, age, group type and size and forage quality. Among all these factors, forage quality may be the most influential factor determining individual changes in time-allocation to different behaviors. For instance, seasonal changes of abovementioned behaviors are tightly linked to forage quality (ADF), but they are also constrained by individual differences in the ability to process and digest forage.

## Supporting information

S1 Fig Proportion of time spent ruminating by bighorn sheep females (open circle) and males (closed square) across seasons estimated using linear mixed models. (DOCX)

S2 Fig Proportion of time spent bedded estimated using linear mixed models a) across seasons by nursery (open circles), male (black squares) and mixed (grey diamonds) groups of bighorn sheep; b) at different ages for each sex. Females = open circles with dashed line; males = black circles with solid line. Age 0 = all lambs less than 1 year of age; c) with an increase in acid detergent fiber (ADF).
(DOCX)

S3 Fig Proportion of bedded time spent ruminating estimated using linear mixed models a) across seasons with data from females = (open circles) and males (closed square); b) at different ages. Age 0 = all lambs less than 1 year of age (females = open circles with dashed line, and males = black circles, solid line); c) with increasing acid detergent fiber (ADF) content in forage.
(DOCX)

S4 Fig Proportion of time spent grazing by bighorn sheep estimated using linear mixed models a) across seasons (females = open circles, and males = closed squares); b) at different ages, separated by sex (females = open circles with dashed line, and males = black circles, solid line). Age 0 = all lambs less than 1 year of age; c) with an increase in acid detergent fiber (ADF) in forage.
(DOCX)
