# Supplementary figures and images for: Effects of intrinsic and extrinsic factors on ruminating, grazing, and bedding time in bighorn sheep (Ovis canadensis)
Source: PLoS One. 2018 Oct 29;13(10):e0206664. doi: 10.1371/journal.pone.0206664 (PMC6205656; doi:10.1371/journal.pone.0206664)

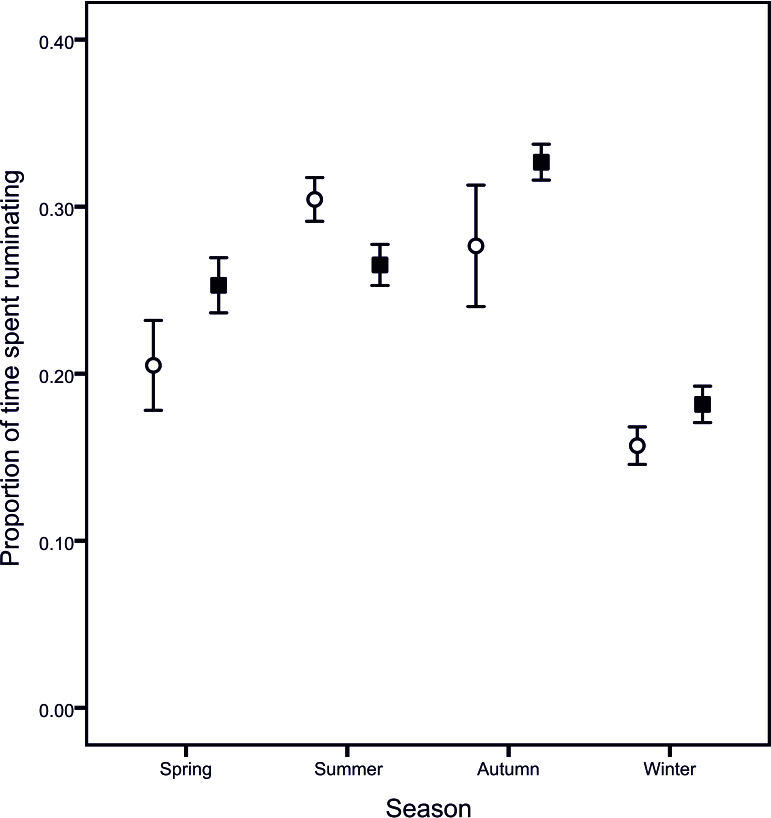

Supplement: S1 Fig — (DOCX) [file pone.0206664.s001.docx]

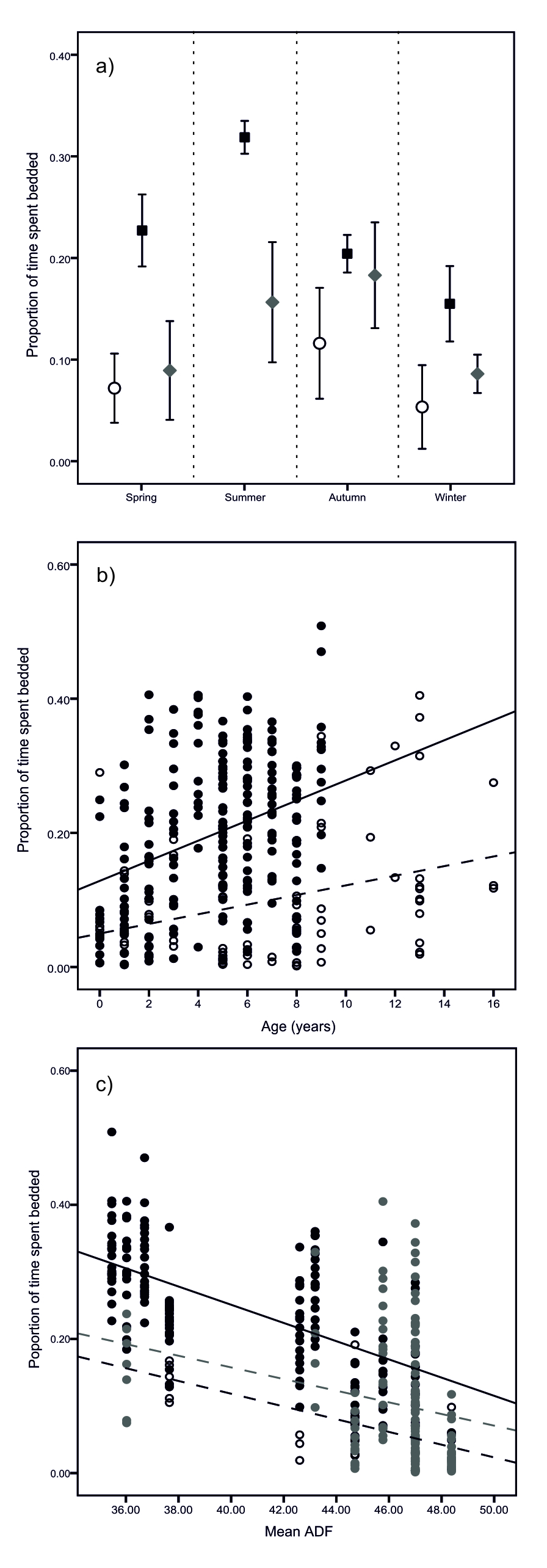

Supplement: S2 Fig — Females = open circles with dashed line; males = black circles with solid line. Age 0 = all lambs less than 1 year of age; c) with an increase in acid detergent fiber (ADF). (DOCX) [file pone.0206664.s002.docx]

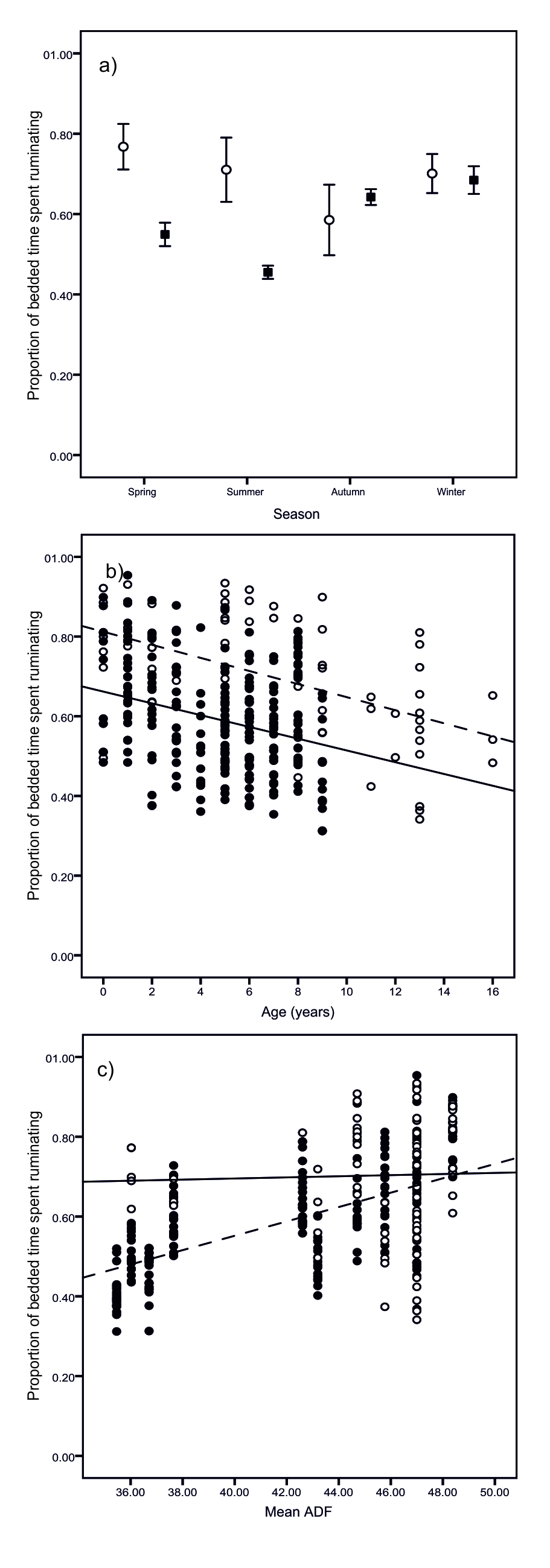

Supplement: S3 Fig — Age 0 = all lambs less than 1 year of age (females = open circles with dashed line, and males = black circles, solid line); c) with increasing acid detergent fiber (ADF) content in forage. (DOCX) [file pone.0206664.s003.docx]

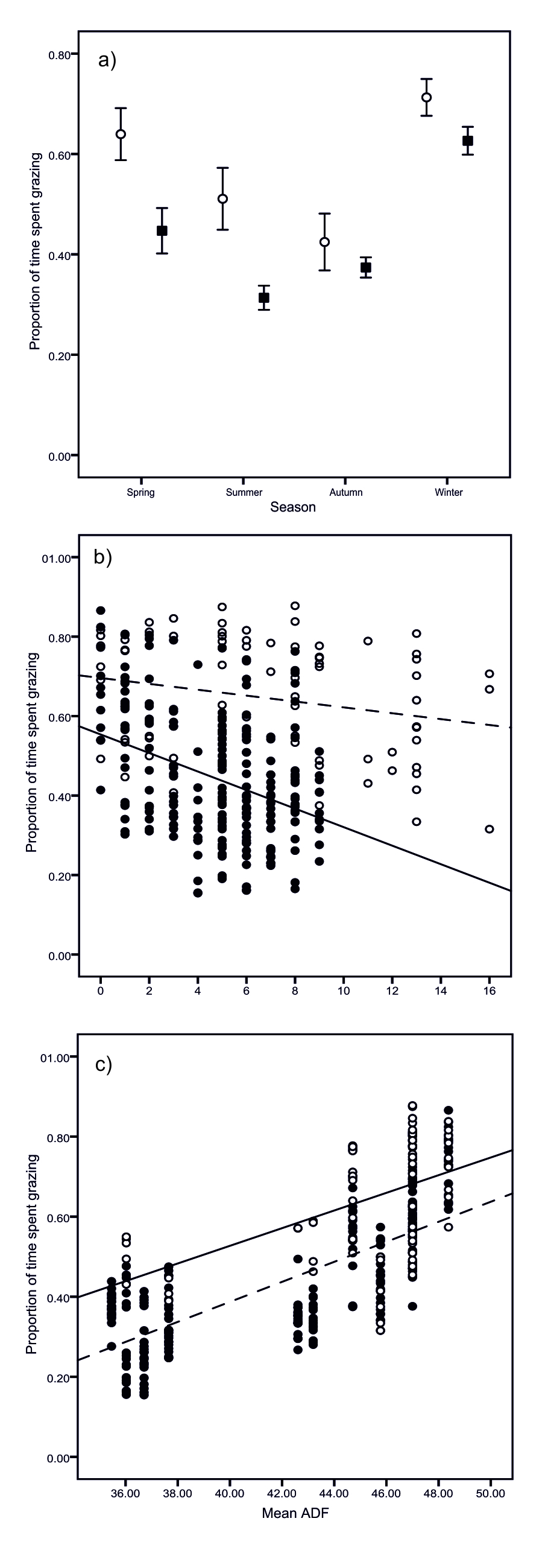

Supplement: S4 Fig — Age 0 = all lambs less than 1 year of age; c) with an increase in acid detergent fiber (ADF) in forage. (DOCX) [file pone.0206664.s004.docx]
